# Tailoring Microstructure and Performance of Cu/SiC Composites via Integrated Powder Metallurgy and Thermo-Compression Processing

**DOI:** 10.3390/ma19020243

**Published:** 2026-01-07

**Authors:** Mohammad Shan, Sajjad Arif, Muhammad Khairi Faiz, Mohd Ridha Muhamad, Ateyah Alzahrani, Ahmad Alghamdi, Anwar Ulla Khan

**Affiliations:** 1 Department of Mechanical Engineering, Faculty of Engineering, University Malaya, Kuala Lumpur 50603, Malaysia; mohd.shan@um.edu.my (M.S.); mkhairifaiz@um.edu.my (M.K.F.); ridha@um.edu.my (M.R.M.); 2 Department of Mechanical Engineering, Faculty of Engineering, Aligarh Muslim University, Aligarh 202002, Uttar Pradesh, India; sajjad.aarif@gmail.com; 3 Centre of Advanced Materials, Faculty of Engineering, University Malaya, Kuala Lumpur 50603, Malaysia; 4 Kagami Memorial Research Institute of Materials Science and Engineering, Waseda University, Tokyo 169-0051, Japan; 5 Centre of Advanced Manufacturing and Materials Processing, Universiti Malaya, Kuala Lumpur 50603, Malaysia; 6 Department of Mechanical and Industrial Engineering, College of Engineering and Computing in Al-Qunfudhah, Umm Al-Qura University, Makkah 715, Saudi Arabia; asalghamdi@uqu.edu.sa; 7 Department of Electrical Engineering Technology, College of Applied Industrial Technology, Jazan University, Jazan 169-0051, Saudi Arabia

**Keywords:** Cu–SiC composites, thermo-compression processing, porosity reduction, grain refinement, microstructure characterization

## Abstract

This study reports the fabrication and characterization of copper–silicon carbide (Cu–SiC) metal matrix composites produced using powder metallurgy (PM) combined with thermo-compression processing (TCP), a dual route that remains limited in Cu–SiC research. Micro-sized SiC particles (1–25 wt.%) were incorporated into Cu, compacted, sintered, and subsequently subjected to sequential forging and annealing. Unlike conventional PM-only processing, TCP significantly reduced porosity, promoted more uniform reinforcement dispersion, and relieved residual stresses, creating a strong synergy between densification and microstructural refinement. SEM, EDS, XRD, and Raman analyses confirmed phase stability, homogeneous reinforcement distribution, and the absence of deleterious interfacial phases. The integrated PM + TCP route achieved an ultimate tensile strength of ~209 MPa, hardness of ~65 HRB, and toughness of ~35 MJ/m^3^ at approximately 3 wt.% SiC. The superior performance at this composition resulted not from the lowest porosity but from the combined effects of uniform particle dispersion, improved particle–matrix bonding, and deformation-driven refinement. These findings establish TCP as an effective post-sintering strategy that overcomes intrinsic porosity and interfacial limitations in Cu–SiC composites. Overall, powder metallurgy combined with the thermo-compression processing is identified as a promising processing pathway for developing high-strength, thermally stable Cu–SiC materials for structural and thermal management applications.

## 1. Introduction

Copper (Cu) and its alloys are widely used in engineering due to their excellent electrical and thermal conductivity. However, their relatively low hardness and poor wear resistance restrict their use in high-load and tribological environments. To address these limitations, various strengthening methods have been applied, including the solid-solution strengthening and the development of copper-based metal matrix composites (Cu–MMCs), which demonstrate superior wear resistance and thermal stability across a wide temperature range [1,2,3,4,5]. Applications, such as cutting tools, brake shoes, and electrical contacts, demand materials that combine high hardness, wear resistance, and thermal conductivity, making Cu–based composites an ideal solution [6,7,8].

Metal matrix composites (MMCs), created by adding ceramic or intermetallic reinforcements to metallic matrices, offer enhanced mechanical and functional properties, including increased strength, reduced density, improved electrical resistivity, and higher thermal stability, compared to unreinforced metals [9,10,11]. The addition of ceramic particles like silicon carbide (SiC), boron carbide (B4C), and alumina (Al_2_O_3_) significantly improves the wear resistance and hardness of aluminum metal matrix composites (Al-MMCs) [12,13,14,15,16]. This is attributed to the high hardness and density of these ceramic particles, which enhance the tribological properties of the composites [17,18,19,20]. Particulate-reinforced MMCs have shown notable enhancements in wear resistance and hardness through the inclusion of ceramic reinforcements like carbides, nitrides, and oxides. Both micro- and nano-scale reinforcements contribute to achieving higher strength-to-weight ratios, enhanced fracture toughness, improved fatigue resistance, and increased corrosion resistance [1,21,22]. TiC and SiC reinforcements enhance MMC toughness, fatigue resistance, and load transfer efficiency [23,24].

In recent years, sustainable methods utilizing industrial and agricultural waste as reinforcement materials have also gained attention in the production of Cu–MMCs, offering a cost-effective approach to enhance mechanical properties and refine the microstructure [25]. Among the various fabrication techniques, powder metallurgy (PM) is commonly employed for Cu–SiC composites due to its near-net-shape ability and reasonable control over reinforcement distribution [26,27]. However, traditional PM-produced Cu–SiC composites often face issues such as porosity, particle agglomeration, and weak interfacial bonding, which reduce their mechanical strength and reliability [21,26,28].

To overcome these limitations, deformation-assisted post-processing methods, such as thermo-mechanical or thermo-compression processing (TCP), have been developed to enhance densification, refine grain structures, and improve particle–matrix bonding [23,29,30,31,32,33]. Previous research has documented various Thermo-compression treatments, including hot rolling at 900–920 °C, followed by ageing [34], cold rolling with intermediate annealing [35], solution treatment and ageing sequences [36], and multi-directional forging with controlled deformation [37], all of which have notably enhanced densification and mechanical performance in metallic systems. SiC-reinforced Al_4_Cu composites show improved strength through thermo-compression processing via extrusion, recrystallization, and particle fragmentation [15,38].

In this study, thermo-compression processing (TCP) has been combined with powder metallurgy to address the porosity and interfacial weaknesses commonly found in traditional Cu–SiC composites. The integrated PM + TCP method improves densification, reduces residual stresses, and refines the microstructure, thereby enhancing the mechanical and structural performance of Cu–SiC metal matrix composites.

## 2. Materials and Methods

### 2.1. Materials

**Figure 1:**
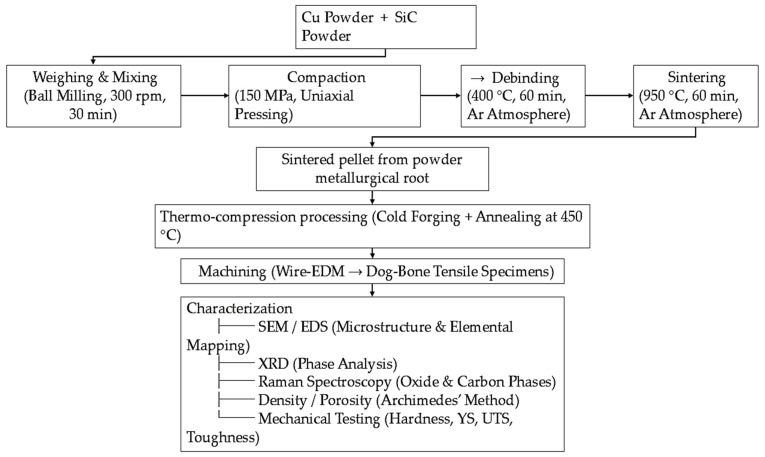
Process flow for Cu–SiC composite fabrication and testing, showing sequential steps from powder preparation to characterization (microstructure, phase, density, and mechanical properties).

The copper (Cu) powder used in this study was obtained from Otto Chemie Pvt. Ltd., Mumbai, Maharashtra, India. The silicon carbide (SiC) powder was sourced from Sarrah Research Laboratories Pvt. Ltd., Andheri (East), Mumbai, India. The Cu powder had a mean particle size of 50 µm, a density of 8.96 g/cm^3^, a melting point of 1356 K, and a molecular weight of 63.5 g/mol. The SiC powder, used as ceramic reinforcement, had a mean particle size of 12 µm, a density of 3.21 g/cm^3^, a melting point of 3003 K, and a molecular weight of 40.11 g/mol. Particle size distribution was determined using a sieve shaker (Fritsch Analysette 3 Pro, Idar-Oberstein, Germany). The overall experimental workflow for fabricating and testing Cu–SiC composites is illustrated in Figure 1, outlining the sequential steps from powder preparation to characterization.

### 2.2. Materials Preparation

Powder mixtures of Cu and SiC were prepared by weighing with a digital balance (Precisa ES 225SM-DR, Dietikon, Switzerland) according to the desired weight fractions shown in Table 1. The powders were blended for 30 min in a low-energy vibratory ball mill (Fritsch Pulverisette MM-1552, Idar-Oberstein, Germany) at 300 rpm, with a ball-to-powder ratio of 10:1, using 10 mm stainless steel balls as the grinding media. To minimize cold welding, 0.5 wt.% stearic acid (C_18_H_36_O_2_) (Otto Chemie Pvt. Ltd., Mumbai, Maharashtra, India) was added during the mixing process [39].

### 2.3. Preparation of Cu–SiC Composites

Cu–SiC powder mixtures were compacted using a uniaxial hydraulic press (Type KE, Sr. No. 1327, Kimaya Engineers, Pune, Maharashtra, India) with a 12 mm steel die at a pressure of 150 MPa and room temperature (25 °C). The walls were lubricated with zinc stearate (C_36_H_70_O_4_Zn) to aid ejection and reduce friction. During pressing, particle rearrangement, deformation, densification, and cold welding occurred, enhancing green strength by increasing contact area and decreasing porosity. Each compact was held under pressure for 30 s to minimize back-pressure effects. To clarify, two different compaction die sizes were used depending on the specimen type. Cylindrical compacts of 12 mm diameter were prepared for density, hardness, and microstructural analyses, while 30 mm diameter compacts were used to fabricate tensile specimens. The powder-mixing, debinding, and sintering parameters were kept identical for both sizes to ensure comparable densification behavior.

**Figure 2:**
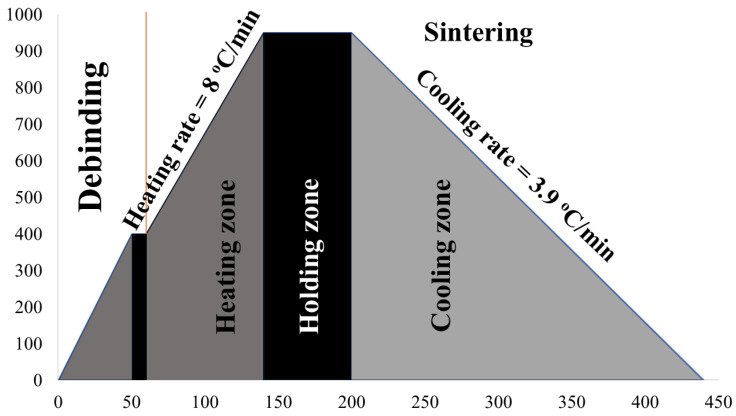
Thermal profile for Cu–SiC sintering showing debinding at 400 °C for 2 min, heating to 950 °C at 8 °C/min, 60 min holding, and natural cooling under argon.

Sintering was conducted in three stages, following a controlled thermal profile shown in Figure 2, in a tubular furnace (Ants Innovations Pvt. Ltd., Bengaluru, Karnataka, India) with a maximum temperature capacity of 1200 °C, under an argon atmosphere (99.999% purity, Inox Air Products Pvt. Ltd., Mumbai, Maharashtra, India). Before the main sintering cycle, a debinding step was introduced to remove stearic acid and zinc stearate by heating the samples to 400 °C and holding for 2 min under argon flow. Stearic acid decomposes almost completely between 175–280 °C, after melting at 68–74 °C, whereas the zinc stearate decomposes above 350 °C with major loss at 400–550 °C, leaving ZnO residue [40]. In the first stage, the samples were heated at a controlled rate of 8 °C/min until they reached the target temperature of 950 °C, where they were held isothermally for 60 min to ensure complete sintering. This stage is shown as the holding zone. In the final stage, natural cooling was employed inside the furnace, with a cooling rate of approximately 3.9 °C/min, until the temperature reached ambient conditions. The cooling rate of the furnace was determined by recording the time required for the temperature drop from the sintering temperature to room temperature.

### 2.4. Thermo-Compression Processing

**Figure 3:**
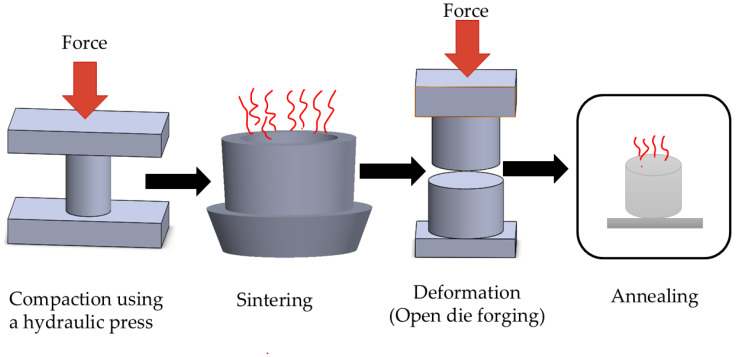
Schematic overview of the thermo-compression processing route, including compaction, sintering, multi-stage deformation, and intermediate annealing applied to Cu–SiC composites.

The thermo-compression process employed in this study consisted of two primary stages: deformation through open-die cold forging and thermal treatment through intermediate annealing. All forging was carried out at room temperature using a hydraulic press under controlled load, while annealing at 450 °C was applied between deformation passes to relieve work-hardening effects and stabilize the microstructure. An overview of the complete processing route encompassing compaction, sintering, multi-stage deformation, and annealing is shown in Figure 3.

**Figure 4:**
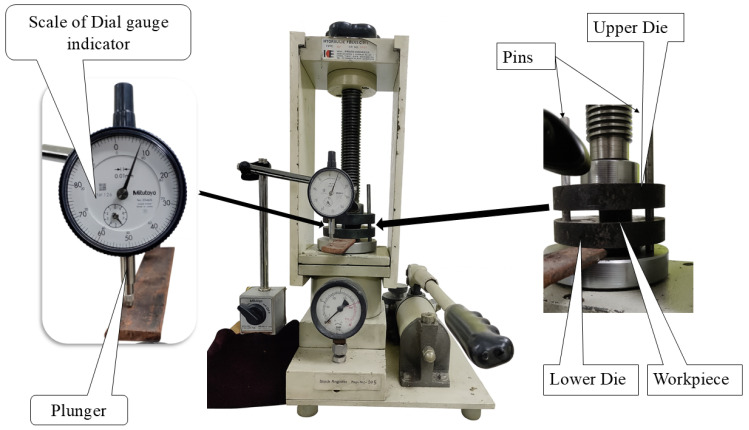
Schematic of the mechanical stage in the Thermo-compression process, showing open die forging under controlled pressure with deformation measured by a dial gauge.

During forging, the specimen was positioned between the upper and lower dies of the press, and a dial gauge (Mitutoyo Corporation, Kawasaki, Japan) was used to monitor height reduction to ensure accurate control of deformation, as shown in Figure 4. Cold deformation enhanced densification and mechanical response, but also introduced residual stresses. These stresses were subsequently reduced through annealing in an argon atmosphere by heating to 450 °C at 8 °C/min, holding for 60 min, and allowing the samples to cool inside the furnace.

A step-wise summary of the thermo-compression sequence is presented in Table 2, detailing the conditions and purpose of each deformation and annealing cycle. Residual stresses, which are stresses remaining in a material without external loads, can be effectively reduced through heat treatments [41].

The degree of deformation was calculated using.

$$Percent\;reduction=\frac{ho-h}{ho}\times 100$$

where ho and h are the initial and final specimen heights. The four consecutive reductions of approximately 20%, 25%, 20%, and 30% resulted in total height reductions of 59–68% for the Cu–SiC composites (Samples A–F), corresponding to a cumulative true strain of 0.9 ± 0.1. Each forging pass was performed with 10–15 s inter-pass intervals to avoid strain localization and maintain uniform densification.

### 2.5. Material Characterization and Evaluation

Morphological analysis of the Cu and SiC powders, as well as the fabricated composites, was carried out using scanning electron microscopy (SEM, Jeol JSM-6510LV, Tokyo, Japan) operated at 15 kV in secondary electron mode. Phase identification was conducted by X-ray diffraction (XRD, Shimadzu XRD-6100, Kyoto, Japan) with Cu Kα radiation (λ = 1.5406 Å), operating at 40 kV and 30 mA. Data were collected over a 2θ range of 10–80° at a scan rate of 0.1°/s and processed using PMGR software (part of PCXRD, Shimadzu Corporation, Kyoto, Japan; version 7.0)). Comparative XRD analyses were performed for pure Cu and Cu–SiC composites. Elemental distribution and reinforcement dispersion were evaluated using energy-dispersive X-ray spectroscopy (EDS) elemental mapping. Raman spectroscopy (Model No. InVia Confocal Make Renishaw, United Kingdom) was performed using a Renishaw inVia spectrometer under atmospheric conditions at 25 °C, with a 532 nm laser, 10% power, ~1 µm spot size, 2400 L/mm grating, spectral range of 102–3002 cm^−1^, 15 s exposure, and five accumulations.

### 2.6. Density and Porosity of Composites

The density of the fabricated composites, referred to as sintered density, is a key parameter for evaluating the efficiency of the sintering process. Measurements were conducted in accordance with ASTM B962-17 [42], utilizing Archimedes’ principle, which involves recording the weight of the sample in air and the apparent weight loss upon immersion in deionized water at 25 °C. A buoyancy correction was applied using the water density at 25 °C (ρ_w_, 25 °C) = 0.9975 g·cm^−3^. The sintered density and percentage porosity were calculated using Equations (1) and (2):

(1) $$Sintered\;Density=\frac{W_{air}}{W_{air}-W_{water}}*\;\rho_{{w,25}^{o}C},$$

(2) $$Porosity\left(\%\right)=\frac{\rho_{th}-\rho_{s}}{\rho_{th}}*100,$$

where w_air_ and w_water_ represent the weights of the specimen in air and in water, respectively, ρ(w,25 °C) = 0.9975 g·cm^−3^ is the water density used for buoyancy correction, ρ_s_ is the measured sintered density (g·cm^−3^), and ρ_th_ is the theoretical density of the composite calculated from the rule of mixtures. Porosity values are expressed in percentage (%). It should be noted that the Archimedes method measures only open and interconnected pores accessible to the immersion medium. Closed porosity, isolated pores trapped within the metallic matrix, is typically underestimated by this method. To distinguish open from closed porosity more accurately, complementary techniques such as helium pycnometry or micro-computed tomography (micro-CT) are often used [40]. In this work, the reported values therefore represent open porosity, while the reduction in closed pores after TCP was inferred from improved densification and SEM observations. The theoretical density, representing the maximum density attainable without voids, was computed using the rule of mixtures based on the composition and pure component densities of copper and SiC [43,44]. The green density, associated with the compacted but unsintered sample, was determined from the measured mass and volume of the green compacts. Green density reflects the initial particle contact area, which promotes bonding during the sintering process [45].

### 2.7. Hardness

The hardness of the composites was evaluated using a digital Rockwell hardness tester (Model TRS-DM, Krutam Techno, Coimbatore, India) in accordance with ASTM E18-17e1 [46]. Measurements were taken at five different points on each sample, and the average value was reported. Testing was performed on the Rockwell B-scale using a 1/16″ steel ball indenter, a significant load of 100 kgf, and a dwell time of 10 s to ensure consistent readings across all specimens [46]. Readings deviating by more than 5% from the mean were treated as outliers and re-measured. The final hardness values represent the average of five replicates (n = 5), with overall variation remaining within ± 2 HRB. All the specimens had an adequate thickness exceeding ten times the indentation depth (≈1.5 mm), thereby satisfying the minimum requirement of ASTM E18 and confirming the absence of substrate influence. As a result, the Rockwell B data reliably represent the bulk hardness of the composites, and Vickers microhardness testing was not required.

### 2.8. Tensile Properties and Data Analysis

**Figure 5:**
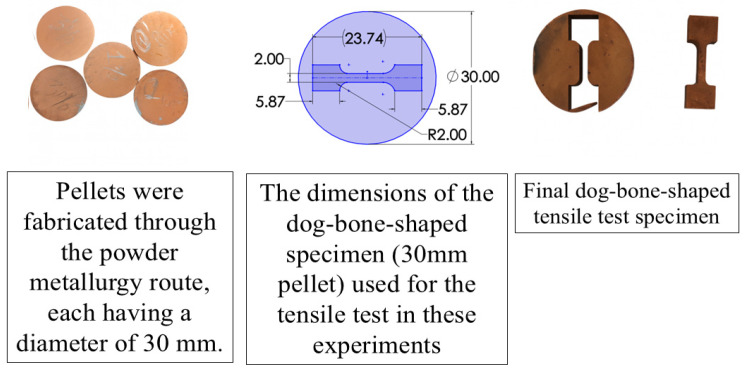
Process sequence from pellet fabrication to final dog-bone shaped tensile specimen prepared using Wire EDM (ELCAM software).

**Figure 6:**
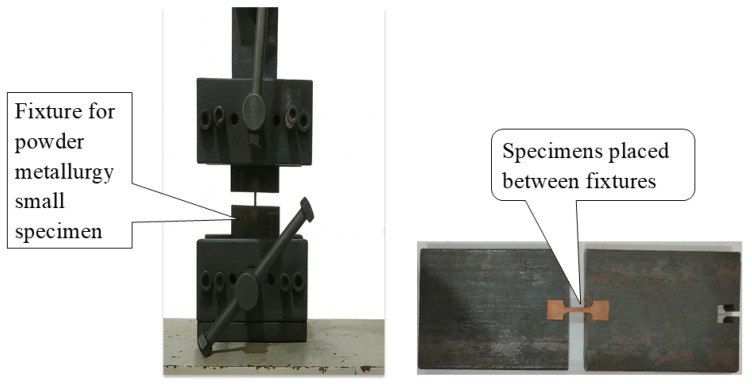
Universal testing machine with a remarkable tensile fixture for securely holding small powder metallurgy pellets during testing.

The initial pellets were produced using a die with a diameter of 30 mm and were subsequently machined into dog-bone-shaped tensile specimens using Wire EDM (ELPULS 50 MARK-2nd, ELECTRONICA, Pune, India). The complete process from pellet fabrication to the final dog-bone specimen is illustrated in Figure 5. The tensile tests were conducted for metallic materials in accordance with ASTM E8/E8M-21 standard [47]. Miniature dog-bone specimens were wire-EDM machined from 30 mm sintered and forged compacts, maintaining a gauge length-to-width ratio of approximately 4:1 and fillet radii of 2 mm, consistent with the standard geometry for sub-size specimens. The specimen thickness was 5 mm, satisfying the ASTM E8/E8M-21 standard dimensional limits for miniature configurations [47]. Testing was conducted on a universal testing machine (UTM-G-410B, Enkay, Calcutta, India) equipped with a specialized tensile fixture designed to hold small powder metallurgy specimens, as shown in Figure 6. The alignment of grips and specimen axes was verified before testing to avoid bending. The crosshead speed of 2 mm min^−1^ corresponded to an initial strain rate of about 1 × 10^−3^ s^−1^, which lies within the prescribed range of ASTM E8/E8M [47] and ISO 6892-1 [48] to maintain uniform strain rate and comparable fracture behavior across all specimens. Although the specimen dimensions were smaller than full-size standards, the geometric ratios and strain rate were preserved; therefore, no size-effect correction was required. All tensile tests were performed on five replicates per composition (n = 5), and the reported ±2% variability corresponds to the standard deviation among these repeated tests.

**Figure 7:**
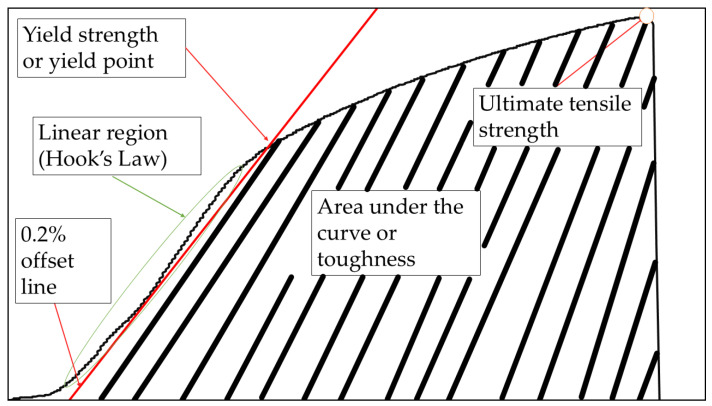
Representative engineering stress–strain curves for Cu and Cu–SiC composites, illustrating variations in elastic modulus, yield strength, ultimate tensile strength, and toughness.

Engineering stress–strain curves for all the key compositions were obtained directly from the UTM load–displacement data and analyzed in accordance with ASTM E8/E8M-21 standard [47]. The yield strength (YS) was defined at 0.2% offset strain, while the ultimate tensile strength (UTS) corresponded to the maximum stress before necking. The toughness (U_t_) was determined as the area under the engineering stress–strain curve up to fracture, representing the total energy absorbed per unit volume during deformation. The integral ($U_{t}=\int_{0}^{\in_{f}}\sigma d\in$) was evaluated numerically using the trapezoidal rule in OriginPro 2023, and the resulting values were expressed in megajoules per cubic meter (MJ m^−3^). These parameters are illustrated in Figure 7, which presents representative stress–strain curves for all studied conditions.

## 3. Results and Discussion

### 3.1. Characterization

#### 3.1.1. Microstructure, SEM, & EDS Mapping

**Figure 8:**
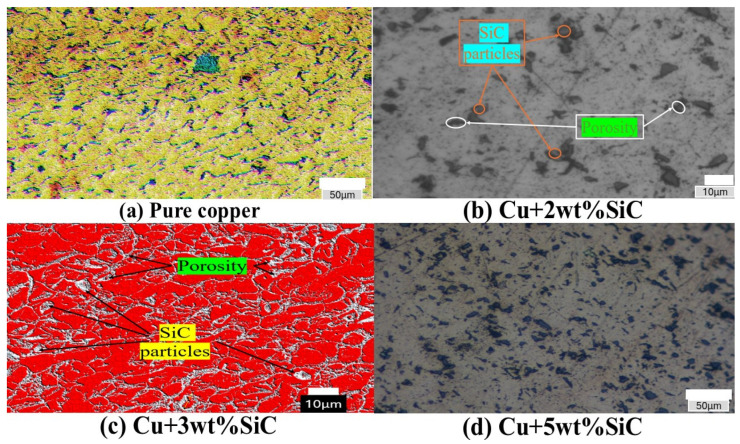
Optical microstructures of Cu/SiC composites after sintering at 950 °C and Thermo-compression processing. (**a**–**d**) Micrographs showing the Cu matrix (light-grey/yellow regions), SiC particles (dark-grey angular shapes), and porosity (black areas). The 3 wt.% SiC composite (**d**) exhibits the most uniform particle dispersion and improved particle–matrix bonding compared to other compositions.

Figure 8 presents the optical microstructures of Cu/SiC composites sintered at 950 °C and subjected to Thermo-compression processing. Within these micrographs, the Cu matrix is identified by yellow, red, and light grey regions in different figures, while the SiC reinforcement component appears as dark grey, angular particles. In Figure 8b,c, the SiC particles are distinctly visible as dark-grey regions, and the black areas correspond to porosity within the composite microstructure. These features reflect the general increase in particle clustering and localized porosity observed with rising SiC content (1–5 wt.%), owing to the limited compressibility and brittleness of SiC. In contrast, the composite containing 3 wt.% SiC exhibits the most uniform particle dispersion and well-bonded interfaces, promoting effective load transfer and densification, as shown in Figure 8c. Achieving this uniform distribution of reinforcement within the matrix is critical for composite materials, as it significantly contributes to the improvement of mechanical, electrical, and thermal properties [49]. Although Figure 8b,c show similar distributions of SiC particles and porosity, their dispersion is less uniform compared to the refined and continuous particle–matrix contact observed in Figure 8d. Figure 8a,c, which were obtained after chemical etching, clearly reveal the grain boundaries of the copper matrix. The etching delineates the microstructural morphology, showing that most grains exhibit irregular or mixed shapes. This morphological diversity of reinforcement particles can influence packing efficiency, interfacial bonding, and load-transfer characteristics, thereby affecting the overall mechanical performance of the composite [50]. Microstructure refinement, improved interfacial bonding, and induced plastic deformation significantly enhance hardness, tensile strength, and structural integrity [51,52,53].

Deformation reduces pore area fraction and average pore size in both pure Cu and Cu–3wt.% SiC, indicating partial pore collapse and improved local densification after thermo-compression. Corresponding SEM images used for the analysis are included in the Supplementary Materials (Figures S1–S4).

**Figure 9:**
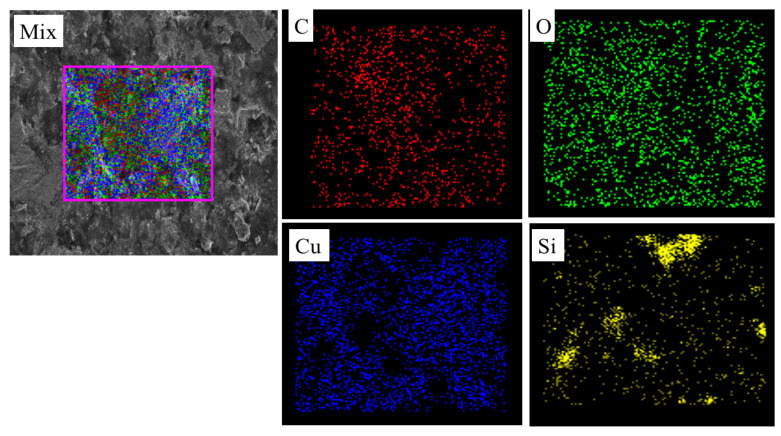
Elemental mapping of the Cu–SiC composite reveals a uniform distribution of Cu, Si, C, and O.

The elemental mapping results obtained from EDS analysis show a well-distributed presence of copper (Cu), silicon (Si), carbon (C), and oxygen (O) within the selected region of the composite sample (refer to Figure 9). The mixed map confirms homogeneous dispersion of all elements across the matrix, with Cu (blue) appearing uniformly throughout, indicating a continuous copper matrix. Si (yellow) and C (red) are dispersed in a particulate manner, corresponding to the presence of SiC reinforcement. A slight non-coincidence is observed between the Si- and C-rich regions, which may result from the surface oxidation of SiC particles and differences in the X-ray interaction volume and detector response for light elements. Umar et al. reported in their study that the presence of O (green) throughout the region suggests minor surface oxidation, likely forming Cu_2_O or CuO phases [54]. According to Efe et al., EDS analysis of Cu–SiC composites sintered at 900 °C and 950 °C revealed an increase in oxygen content with rising sintering temperature and SiC weight percentage, particularly at the Cu–SiC interface and on smaller SiC particles [55].

**Figure 10:**
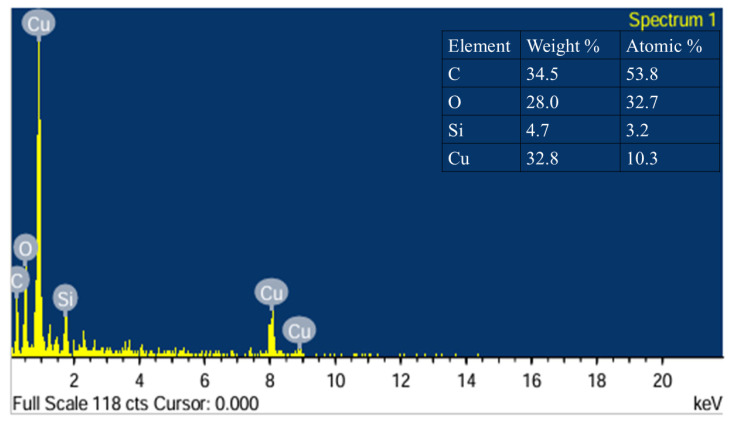
EDS spectrum of the Cu–SiC composite showing characteristic peaks of Cu, Si, and C elements, confirming the presence and uniform distribution of SiC reinforcement within the copper matrix.

The EDS spectrum confirms the elemental composition of the Cu–SiC composite, showing the presence of carbon (C), oxygen (O), Silicon (Si), and copper (Cu) (refer to Figure 10). The quantitative analysis indicates a high carbon content at 34.5 wt% (53.80 at%), which likely originates from the SiC reinforcement and possible surface contamination. Oxygen is present at 28.01 wt% (32.77 at%), suggesting surface oxidation of copper, possibly forming Cu_2_O or CuO. Silicon, present at 4.72 wt% (3.15 at%), corresponds to the SiC phase, verifying the successful incorporation of reinforcement. Copper constitutes 32.77 wt% but only 10.28 at%, consistent with its higher atomic mass. These results agree with the elemental mapping and indicate a well-dispersed composite system. Rohbeck et al. reported that SiC oxidation forms a surface SiO_2_ layer in oxygen atmospheres, increasing oxygen content, while high-temperature decomposition releases silicon vapor, leaving excess free carbon that raises the carbon content [56].

#### 3.1.2. X-Ray Diffraction Spectroscopy (XRD)

**Figure 11:**
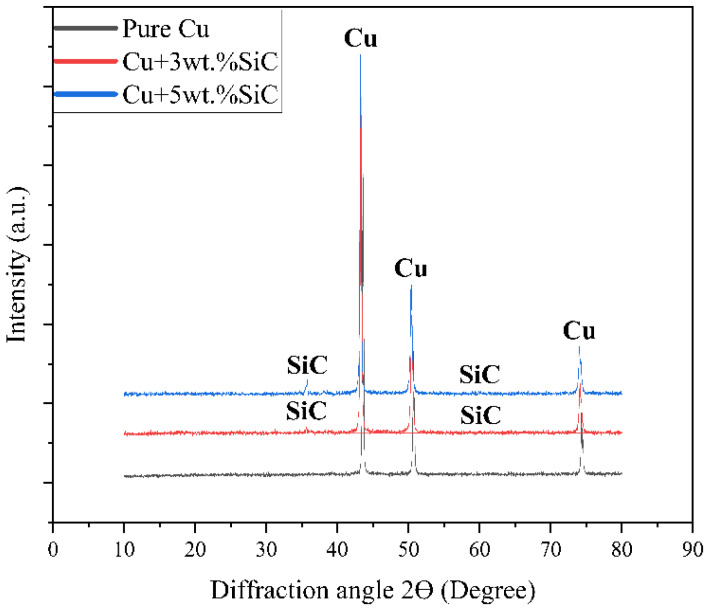
XRD patterns of samples pure copper, Cu–3wt.% SiC, and Cu–5wt.% SiC showing diffraction peaks for face-entered cubic Cu and SiC phases.

Figure 11 presents the XRD patterns of pure Cu and Cu–SiC composites (3 and 5 wt.% SiC) fabricated by powder metallurgy followed by thermo-compression. The diffraction peaks at 2θ ≈ 43.3°, 50.4°, and 74.1° correspond to the (111), (200), and (220) planes of FCC copper (JCPDS 04-0836), while additional peaks near 36°, 60°, and 72° are indexed to hexagonal SiC (JCPDS 29-1129), confirming phase stability without formation of Cu_2_O, CuO, or Cu–Si compounds. Comparison of the relative intensities of Cu peaks yielded texture coefficients close to unity (TC_(111)_ = 1.05, TC_(200)_ = 0.97, TC_(220)_ = 0.98), indicating negligible preferred orientation after the thermo-compression. No secondary phases were detected within the XRD detection limit of approximately 2–3 wt.%, verifying chemical compatibility and uniform phase retention between Cu and SiC after processing. Slight variations in peak intensity, particularly for SiC, may be due to differences in reinforcement distribution or orientation during processing [55].

#### 3.1.3. RAMAN Spectroscopy

**Figure 12:**
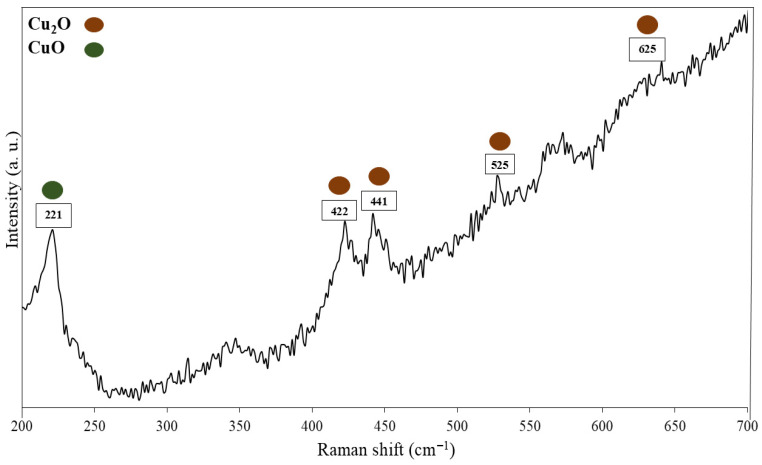
Raman spectrum of the copper.

The Raman spectrum exhibits distinct peaks at 221, 422, 441, 525, and 625 cm^−1^, indicating surface oxidation of the copper sample. The peak at 221 cm^−1^ corresponds to CuO, while the remaining peaks are attributed to second-order Raman modes of Cu_2_O, as shown in Figure 12. These results suggest the formation of a thin oxide layer on the copper surface, likely due to exposure to ambient air during handling or storage [57].

**Figure 13:**
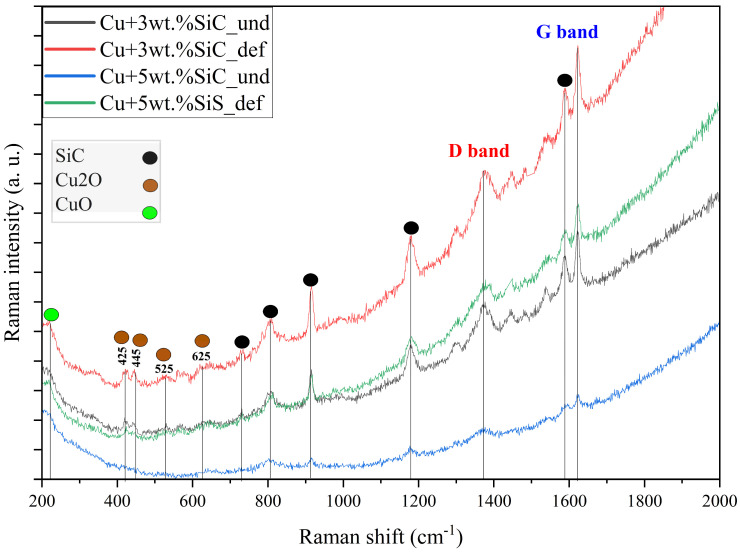
Raman spectra of Cu–SiC composites.

Figure 13 illustrates that the Raman spectra of Cu–SiC composites (Cu–3wt.% SiC_und, Cu–3wt.% SiC_def, Cu–5wt.% SiC_und, Cu–5wt.% SiC_def) exhibit key peaks at 221, 445, 525, and 625 cm^−1^, corresponding to CuO and second-order modes of Cu_2_O, confirming surface oxidation of the copper matrix likely due to ambient exposure. Broad peaks between 780 and 1200 cm^−1^ are attributed to Si–C vibrational modes, indicating the presence and structural integrity of SiC reinforcement in all samples. The Raman spectra of Cu–SiC composites exhibit two distinct bands at ~1350 cm^−1^ (D) and ~1580 cm^−1^ (G), corresponding to the disordered and graphitic carbon phases, respectively. These features are absent in the standard SiC spectrum, which shows strong phonon modes at ~796 cm^−1^ (TO) and ~973 cm^−1^ (LO) for 3C-SiC and additional weaker bands near 789 cm^−1^ and 967 cm^−1^ for 6H-SiC [58]. The appearance of the D (~1350 cm^−1^) and G (~1580 cm^−1^) bands in the Raman spectra of the Cu–SiC composites, and their increased intensity after deformation, can be explained by several interconnected mechanisms. Partial interfacial decomposition of SiC during sintering at 950 °C may release small amounts of sp^2^-bonded carbon at particle boundaries, especially in regions where oxygen is present, contributing to both bands. Deformation processing further increases disorder at the Cu/SiC interface by fracturing SiC edges and generating defect-rich carbon species, consistent with SEM evidence of particle fragmentation after forging. Although surface contamination can also generate D and G features, the absence of C–H (2800–3100 cm^−1^) and C=O (~1700 cm^−1^) stretching peaks, together with spectral reproducibility under 532 nm and 785 nm excitation (<2 mW), makes this explanation unlikely. Yuan et al. reported that the D (~1350 cm^−1^) and G (~1580 cm^−1^) Raman bands arise from sp^2^-bonded carbon structures: the D band reflects breathing modes of disordered aromatic rings, while the G band corresponds to stretching of sp^2^ carbon pairs. Their intensity ratio indicates the degree of graphitization, disorder, and crystallite size in carbon materials [59]. Deformation breaks brittle SiC particles, producing microcracks, defect-rich carbon, and dislocations at the Cu/SiC interface, increasing structural disorder and altering local mechanical behavior [60]. Shtepliuk et al. similarly reported SiC fragmentation, copper oxidation effects, and processing-induced structural changes, confirming the deformation-driven evolution observed in this study [61].

### 3.2. Deformation

**Figure 14:**
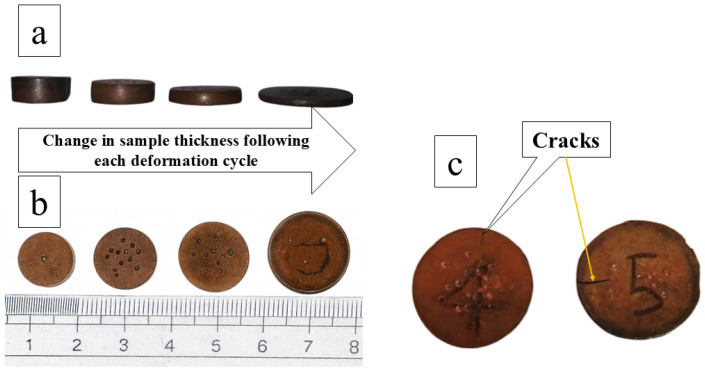
Cu/SiC composite pellets after each deformation stage, (**a**,**b**) showing increased diameter and reduced thickness due to volume constancy. (**c**) shows surface cracks appearing in the pellet, indicating over-deformation.

The deformation behaviour of the Cu–SiC composites was evaluated through four sequential open-die cold forging steps (Deform 1–Deform 4), with intermediate annealing at 450 °C used to relieve work hardening and restore ductility. Each forging pass reduced specimen height and increased diameter according to the principle of volume constancy in cold working [62]. The dimensional changes after each deformation stage are shown in Figure 14, where excessive deformation produces surface cracks, as seen in Figure 14c.

**Figure 15:**
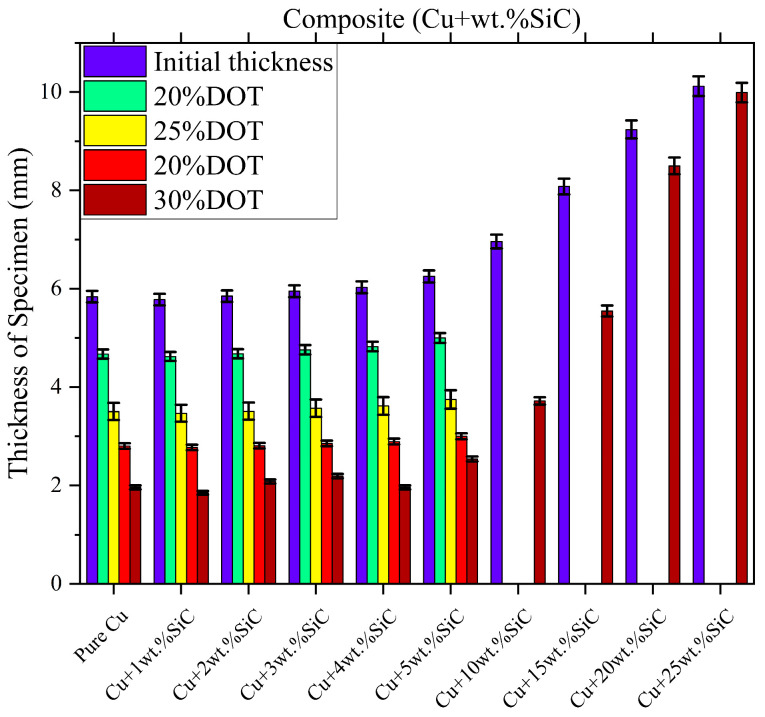
Maximum deformation (%) of Cu–SiC composites as a function of SiC content, with ±2% error bars representing experimental variation (n = 5).

The corresponding specimen heights and total deformation percentages are listed in Table 3, shown in Figure 15, revealing a clear relationship between SiC content and deformability. Pure Cu and low-SiC composites (1–5 wt.% SiC) exhibit high deformation capacities of 59–68%, enabled by the ductile copper matrix and relatively uniform particle distribution. In contrast, higher SiC contents (10–25 wt.%) substantially reduce deformation, with total reductions falling below 50% at 10 wt.% SiC and becoming minimal at 20–25 wt.% SiC due to increased brittleness, particle agglomeration, and limited matrix plasticity.

The final deformation stage represented the maximum safe reduction achievable before crack initiation. Overall, the multistep deformation–annealing sequence provides controlled densification and accommodates varying reinforcement levels, enabling systematic adjustment of the composite’s plastic response.

### 3.3. Physical Properties

#### 3.3.1. Density

Table 4 shows that sintered density decreases and porosity increases with rising SiC content, due to the lower density and limited compressibility of SiC particles. The Cu–3wt.% SiC composite exhibits relatively high density (8.0 g cm^−3^) and moderate porosity (15.2%), indicating better particle rearrangement and bonding. Excess reinforcement (>3 wt.%) hinders densification, leading to higher residual porosity.

**Figure 16:**
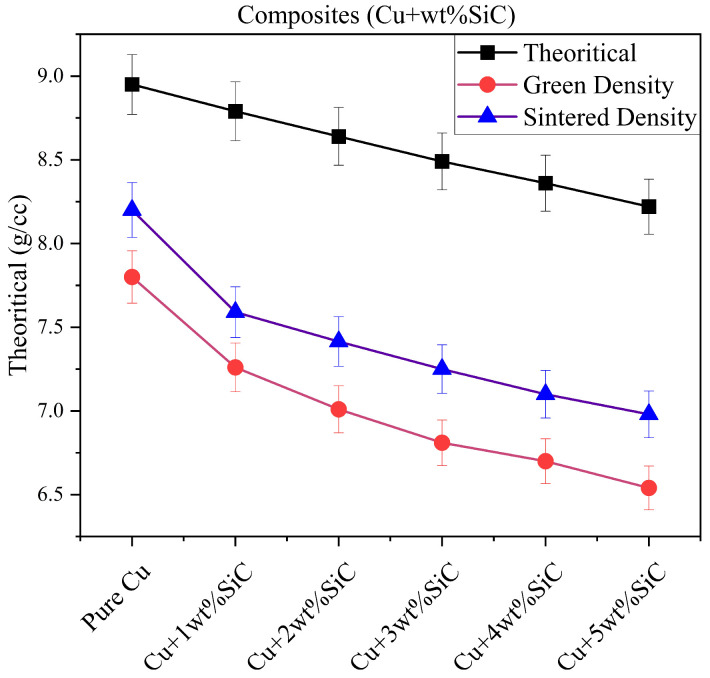
Theoretical, green, and sintered densities of Cu–SiC composites with ±2% error bars representing experimental variation (n = 5).

Figure 16 presents the variation in theoretical, green, and sintered densities of Cu–SiC composites as a function of increasing SiC content, with ±2% error margins included for each dataset. The theoretical density decreases consistently with reinforcement due to the lower density of SiC relative to copper. The maximum relative green density of 82.59% is recorded for the Cu + 1 wt.% SiC composite, while pure copper shows the lowest value of 79.44%. The slight improvement with 1 wt% SiC is within the uncertainty range, yet it indicates better particle packing, where the reinforcement disrupts uniform copper stacking and reduces voids. A comparable trend is observed for sintered densities, where Cu + 1 wt.% SiC reaches the highest relative density of 86.35%, while pure copper remains at 80.45%, confirming enhanced bonding and shrinkage. At higher SiC contents, both green and sintered densities exhibit systematic decreases beyond the error margins, reflecting the development of porosity and poor rearrangement during compaction, which is linked to the hardness and angular morphology of SiC. Post-deformation relative density further emphasizes this trend: pure copper achieves 97.35% after Thermo-compression processing, whereas Cu + 5 wt.% SiC declines to 92.27%. These findings are consistent with microstructural evidence of particle clustering and interfacial voids. The data confirm that low SiC additions can aid densification, while excessive reinforcement increases porosity and hinders consolidation. Thus, approximately 1 wt.% SiC is identified as the most effective level for maintaining compaction efficiency and sintering performance in Cu–SiC composites [49].

#### 3.3.2. Density Variation with the Thermo-Compression Process

**Figure 17:**
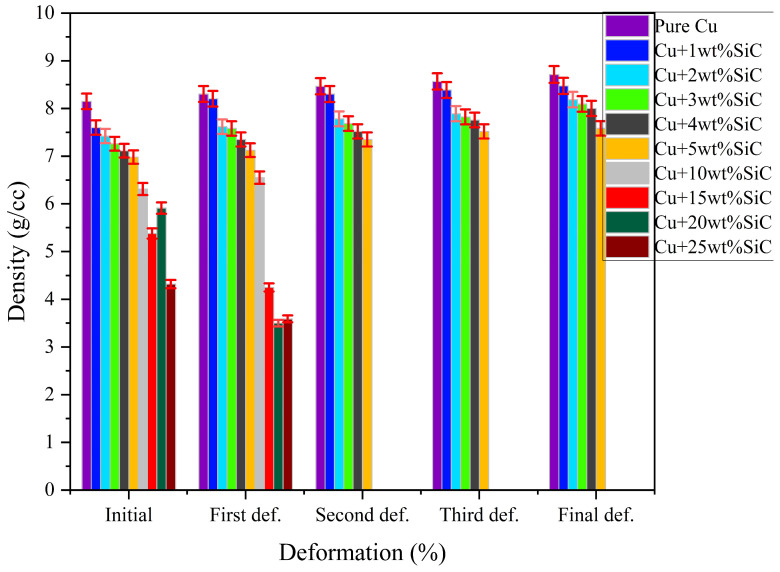
Variation in density of Cu–SiC composites with deformation stage, showing progressive densification, with ±2% error bars representing experimental variation (n = 5).

Figure 17 illustrates the evolution of density in Cu–SiC composites (from pure Cu to Cu + 25 wt.% SiC) across sequential deformation stages, from the initial compact to the final deformation operation. A clear trend of increasing density is observed with each deformation stage, attributed to the enhanced packing and reduction in porosity that occur because of plastic deformation. Notably, samples with lower SiC content (Cu to Cu–25wt.% SiC) exhibit more significant densification gains, reaching values above 8.5 g/cm^3^ in the final stage. In contrast, higher SiC-containing composites (Cu–5wt.% SiC to Cu–25wt.% SiC) show relatively moderate increases. This disparity is primarily due to the rigid and brittle nature of SiC particles, which hinders particle rearrangement and restricts plastic flow during forging. Furthermore, composites with higher SiC content initially possess greater porosity due to poor compatibility and particle agglomeration, as confirmed by microstructural analysis. As deformation progresses, strain-induced densification occurs more efficiently in ductile copper-rich matrices, while the efficiency diminishes in brittle-rich systems. The plotted data include ±2% error bars, indicating minimal scatter and good repeatability of the density measurements. The final density profiles indicate that Thermo-compression processing significantly enhances structural integrity, particularly in lower reinforcement composites, by reducing voids and promoting improved interfacial bonding. However, an optimal balance between reinforcement content and process strain is crucial to maximize densification without inducing premature cracking or compromising composite stability. Wąsik et al. reported that the density of the Al_4_Cu–SiC composites slightly decreased with increased SiC content due to particle cracking and residual porosity formed during hot extrusion and sintering [15].

#### 3.3.3. Porosity

**Figure 18:**
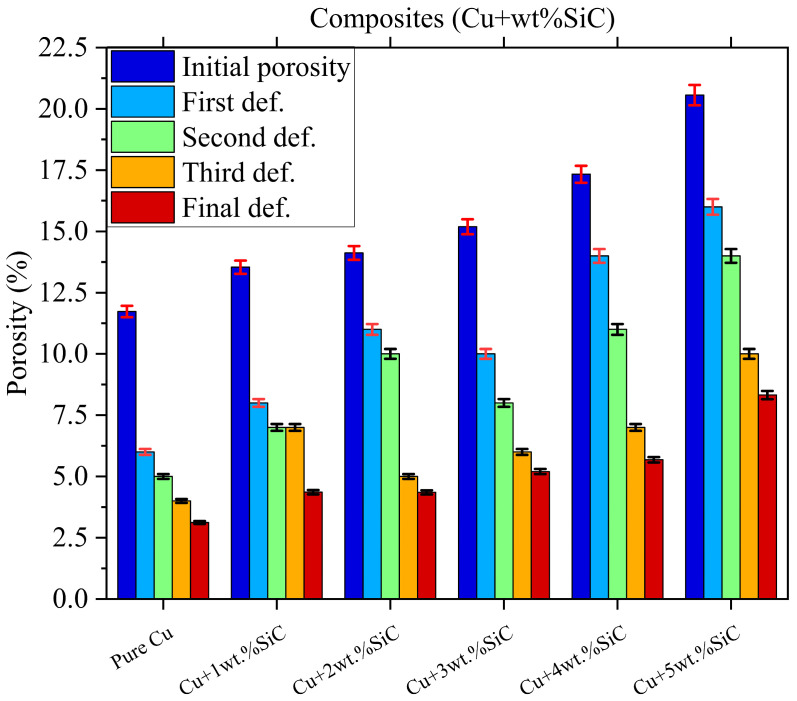
Porosity reduction in Cu–SiC composites across successive deformation stages, with ±2% error bars indicating experimental variation (n = 5).

Figure 18 illustrates the reduction in porosity of Cu–SiC composites (Cu to Cu–25wt.% SiC) across progressive deformation stages, highlighting the beneficial impact of Thermo-compression processing on densification. Initially, porosity levels are higher in composites with increased SiC content, as seen in Samples Cu + 4 wt.% SiC and Cu + 5 wt.% SiC, due to the inherent brittleness, poor compressibility, and particle agglomeration associated with ceramic reinforcements. However, with each deformation stage, ranging from the first to the final deformation, a clear and consistent decline in porosity is observed across all samples. This can be attributed to the application of compressive stress during open-die forging, which facilitates pore closure and enhances interparticle contact. The plotted data include ±2% error bars, confirming the reproducibility and accuracy of the porosity measurements. Notably, lower SiC-content composites (Cu–Cu + 2 wt.% SiC) exhibit greater porosity reduction efficiency, achieving final porosity values below 5%, which indicates better plastic flow and deformation compatibility with the copper matrix. In contrast, a higher SiC content limits plastic deformation and impedes full pore closure, resulting in relatively higher residual porosity despite multiple deformation steps. These observations align with microstructural analyses that reveal improved particle packing and reduced voids in ductile-rich matrices. Overall, the data emphasize that while Thermo-compression processing significantly mitigates porosity, the effectiveness diminishes with increasing ceramic reinforcement, necessitating careful optimization of SiC content to achieve both mechanical enhancement and structural integrity [2,15].

#### 3.3.4. Rockwell Hardness

**Figure 19:**
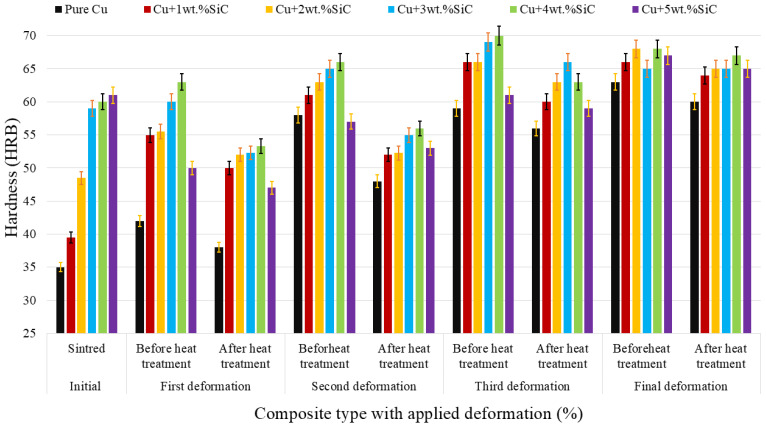
Variation in hardness (HRB) of Cu–SiC composites across sintering, deformation, and heat-treatment stages, with ±2% error bars representing experimental variation (n = 5).

Figure 19 illustrates the evolution of hardness in Cu–SiC composites (Cu–Cu + 5 wt.% SiC) across different stages of deformation and heat treatment. A clear upward trend is observed in hardness values with increasing deformation, attributed primarily to strain hardening induced during cold working. The highest hardness values are recorded after the third and final deformation stages, where densification and microstructural refinement are maximized. Additionally, composites with higher SiC content (e.g., Composites Cu + 4 wt.% SiC and Cu + 5 wt.% SiC consistently exhibit superior hardness across all stages due to the intrinsic hardness of SiC and its effective load-transfer role within the copper matrix.

The application of intermediate annealing after each deformation step results in a temporary reduction in hardness, reflecting the partial recovery and recrystallization processes that relieve internal stresses. However, the post-annealing hardness values remain higher than those of the preceding stage, indicating that cumulative work hardening has occurred. This alternating pattern between deformation and heat treatment highlights the efficiency of Thermo-compression processing in achieving a balance between strength and ductility. The results correlate with microstructural observations, which show increased particle-matrix bonding and grain refinement, particularly in samples with moderate SiC content. The plotted data include ±2% error bars, confirming good reproducibility and minimal experimental scatter. Overall, the data confirm that the combined effects of reinforcement, deformation, and thermal cycles synergistically enhance the hardness of Cu–SiC composites, with optimized gains in samples processed under controlled conditions. Sharma et al. reported that the hardness of the composite significantly improved following the re-pressing treatment, approaching the values typically reported for oxygen-free high-conductivity (OFHC) copper produced through the melting and refining of electrolytic copper slabs [63].

### 3.4. Mechanical Properties

#### 3.4.1. Yield Strength

**Figure 20:**
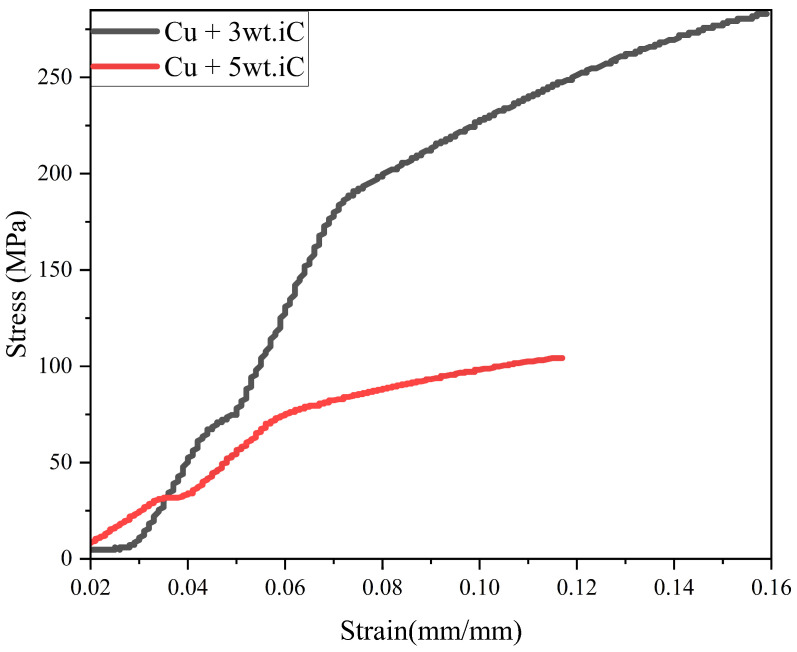
Stress—strain diagram of copper and SiC composites.

Table 5 summarizes the yield strength (YS) of Cu–SiC composites before and after the deformation. The addition of SiC reinforcement increases YS compared to pure Cu (45.5 MPa), reaching a maximum of 107.4 MPa in the undeformed Cu–1wt.% SiC composite due to effective load transfer and grain-boundary strengthening. After the deformation, a slight reduction in YS is observed, particularly in higher SiC-loaded samples. This occurs because local stress relaxation and partial recovery of dislocations offset the strengthening gained from densification. As shown in Figure 20, the Cu–3wt.% SiC composite maintains uniform deformation, while Cu–5wt.% SiC exhibits early deviation from linearity and reduced work-hardening capacity. This indicates that excessive reinforcement limits plastic flow and promotes localized deformation despite improved density. Therefore, moderate SiC additions (1–3 wt.%) provide the best balance of strength and structural stability.

#### 3.4.2. Ultimate Tensile Strength

The ultimate tensile strength (UTS) values in Table 5 show a consistent improvement for all Cu–SiC composites after Thermo-compression processing. Pure Cu increased from 71 MPa to 146 MPa due to combined densification and strain hardening, while Cu–1wt.% SiC and Cu–3wt.% SiC reached 193 MPa and 209 MPa, respectively. Unlike the YS, UTS continues to rise because improved particle–matrix bonding, reduced porosity, and enhanced dislocation density raise the load-bearing capacity before fracture. The Cu–3wt.% SiC composite shows near-uniform particle dispersion, ensuring efficient stress transfer and steady strain hardening, while Cu–5 wt.% SiC displays lower UTS (147 MPa) due to particle clustering and restricted matrix flow. Thus, deformation enhances the overall tensile strength, even if minor softening occurs at the yield stage, with the optimum reinforcement level around 3 wt% SiC. Somani et al. reported that adding 20% SiC to copper increased hardness by 48% and tensile strength by 24%, significantly improving wear resistance, as evidenced by a 45–77% reduction in wear rate, and lowering friction by 25–44% [64].

#### 3.4.3. Toughness

Toughness, obtained from the area under the stress–strain curve, reflects the material’s ability to absorb energy before fracture. As shown in Table 5, toughness increases with SiC addition up to 3 wt.% in the undeformed condition, reaching 25.6 MJ m^−3^, about ten times that of pure Cu—indicating effective load sharing between matrix and reinforcement. After deformation, toughness further improves, reaching 35.9 MJ m^−3^ and 35.1 MJ m^−3^ for Cu–1wt.% SiC and Cu–3wt.% SiC, respectively. This improvement arises from densification, grain refinement, and stronger interfacial bonding, which delay the crack initiation; however, at 5 wt.% SiC, toughness drops to 8.8 MJ m^−3^ due to restricted plasticity and increased brittleness. Overall, moderate reinforcement and controlled deformation provide the best synergy of strength, ductility, and fracture resistance in Cu–SiC composites.

#### 3.4.4. Combined Effect of Density and Porosity on UTS and Hardness

**Figure 21:**
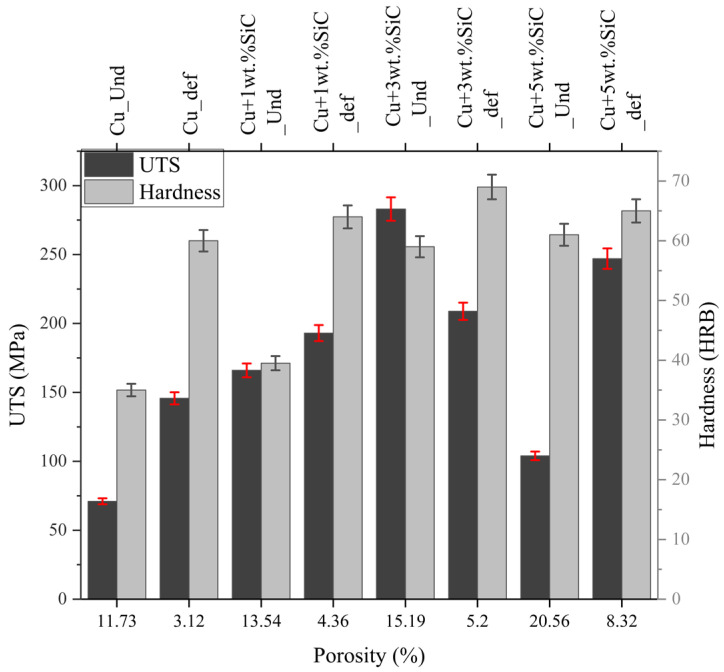
UTS and hardness vs. porosity for undeformed and deformed Cu–SiC composites, with ±2% error bars representing experimental variation.

The combined analysis of UTS and hardness as a function of porosity (Figure 21) and density (Table 5) demonstrates a clear improvement in mechanical properties following Thermo-compression processing. Deformed samples consistently show lower porosity (2.08–6.26%) and higher density (7.58–8.71 g/cm^3^) compared to their undeformed counterparts, which directly contributes to enhanced UTS and hardness through pore closure, improved particle–matrix contact, and strain-induced grain refinement. For example, pure Cu shows an increase from 71 → 145.7 MPa (UTS) and 35 → 60 HRB (hardness) as porosity decreases from 11.73% to 3.12% and density increases from 8.15 → 8.71 g/cm^3^. A similar densification-driven improvement is observed in the Cu + 1 wt.% SiC composite, where UTS increases from 166 → 193 MPa and hardness from 39.5 → 64 HRB as porosity decreases from 13.54% to 4.36% and density rises from 7.60 → 8.47 g/cm^3^. In the 3 wt.% SiC composite, the undeformed condition shows the highest UTS (283 MPa) at a porosity of 15.19% and density of 7.26 g/cm^3^, due to favorable SiC dispersion despite higher residual porosity. After deformation, UTS decreases to 208.8 MPa, but hardness increases to 69 HRB as porosity reduces to 5.2% and density improves to 8.09 g/cm^3^. These results confirm that the enhanced mechanical performance of the 3 wt.% SiC composite is not due to possessing the lowest porosity, as previously stated, but rather due to the combined effects of densification, uniform reinforcement distribution, and strengthened particle–matrix interfaces. The 5 wt.% SiC composite shows the strongest correlation between porosity and performance: in the undeformed state, high porosity (20.56%, density 6.98 g/cm^3^) leads to low UTS (104 MPa), whereas deformation significantly improves density (7.58 g/cm^3^), reduces porosity (8.32%), and raises UTS to 247 MPa with hardness increasing to 65 HRB.

Overall, the data demonstrate that increased density and reduced porosity consistently enhance UTS and hardness across all compositions, but the magnitude of improvement also depends on reinforcement dispersion and deformation-induced microstructural refinement, not porosity alone. This corrected interpretation aligns fully with the plotted values in Figure 21 and Table 5. Conversely, increased porosity weakens mechanical integrity, lowering Young’s modulus, shear strength, and fatigue resistance due to stress concentration and crack initiation [65].

#### 3.4.5. Fractography Analysis

**Figure 22:**
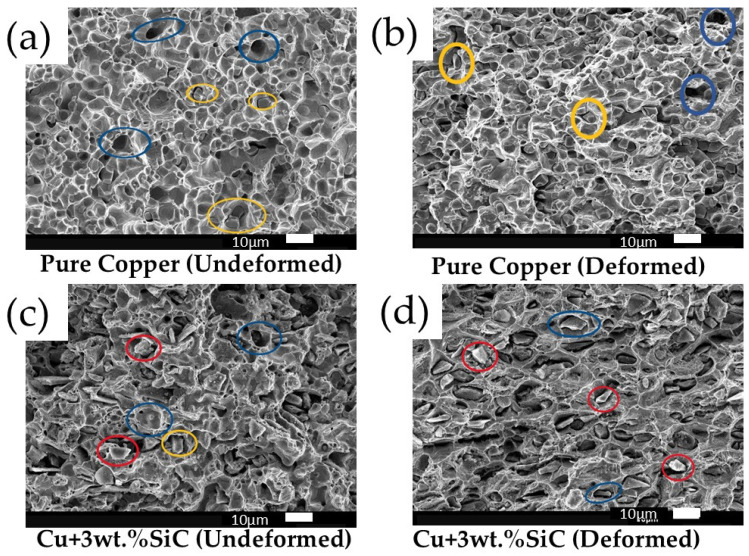
SEM fractography of (**a**) Pure Cu (undeformed), (**b**) Pure Cu (deformed), (**c**) Cu–3wt.% SiC (undeformed), and (**d**) Cu–3wt.% SiC (deformed). Undeformed samples show deep equiaxed dimples, while deformation produces elongated dimples, reduced pore size, and improved particle–matrix bonding. Scale bar: 10 µm.

The SEM fractography in Figure 22a–d shows the fracture behavior of pure copper and Cu–3wt.% SiC composites under undeformed and deformed conditions. Figure 22a represents the undeformed pure copper sample, while Figure 22b corresponds to the thermo-compression-processed (deformed) pure copper specimen. Figure 22c,d show the undeformed and deformed Cu–3wt.% SiC composites, respectively. Blue circles highlight voids and dimples of varying sizes, indicating ductile fracture in the copper matrix, while yellow circles mark fractured SiC particles, confirming their brittle nature. Red circles show intact or partially deboned SiC particles. The thermo-compression processing yields more refined and uniformly distributed SiC particles compared to the larger, clustered ones found in undeformed samples. This particle refinement, along with improved grain structure and matrix bonding, enhances tensile properties and fracture resistance of the composite. Multiple studies have demonstrated that reinforcing metal matrices with particles such as SiC, steel, Mo, or Ti enhances strength, hardness, and fracture toughness. While steel and Mo reinforcements offer better ductility due to their inherent deformability, SiC often leads to brittle fractures [22,64].

**Figure 23:**
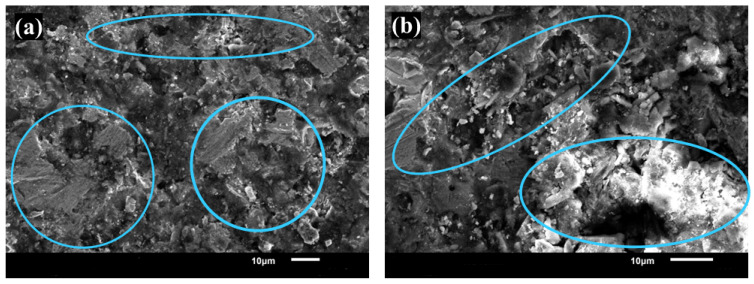
SEM fractography showing mixed-mode fracture in Cu + 5 wt.% SiC composites. Illustrate the transition from ductile to brittle behavior with increasing SiC content. (**a**) Lower-magnification 800X (**b**) Higher-magnification 1500X.

The fracture behavior of Cu–SiC composites is predominantly brittle of the composite (Cu + 5 wt.% SiC), governed by the inherent brittleness of the SiC reinforcement. Fractography reveals random cracking, with most of the fractured surface being relatively flat. The blue circles in Figure 23 highlight flat fracture surfaces and randomly distributed cracks. With increasing SiC content, the extent of brittle cracking intensifies, limiting plastic deformation and leading to premature failure, as illustrated in Figure 23. This results in higher stress concentrations and poor load transfer, ultimately resulting in a decline in mechanical properties. Consequently, UTS, YS, elongation, and toughness decrease with increasing SiC content [66].

### 3.5. Oxidation Effects Discussion

The presence of Cu_2_O and CuO detected through XRD, Raman spectroscopy, and EDS mapping has important implications for mechanical performance and fracture behaviour. These oxides tend to form preferentially along particle boundaries and pore surfaces during sintering. They may act as brittle interfaces that reduce load-transfer efficiency between the copper matrix and SiC reinforcement. Cu_2_O is known to degrade ductility by promoting micro-void formation during deformation, while CuO can further embrittle the matrix due to its higher brittleness and poor interfacial cohesion with metallic copper. Studies show that the formation of Cu_2_O and CuO during the sintering of copper matrix composites significantly impacts their mechanical properties by reducing ductility and strength through the promotion of micro-void formation and poor interfacial cohesion. Optimizing sintering conditions and material selection are crucial strategies to mitigate these effects and enhance the performance of the composites [67,68]. The fractography of undeformed and deformed samples supports this interpretation: regions containing oxide films exhibit cleavage-like features and particle pull-out, indicating weakened interfacial bonding. In contrast, areas with reduced oxide content show ductile dimples and improved matrix continuity. Thus, even small amounts of oxidation contribute to localized embrittlement, reduced strain accommodation, and premature cracking, thereby influencing both the tensile response and the overall fracture mode of the Cu–SiC composites. Similar trends observed in previous studies oxidation in Cu–SiC composites promotes Cu_2_O/CuO formation, weakening interfacial bonding and causing cleavage-like fracture, particle pull-out, and localized embrittlement. These brittle oxide films reduce strain accommodation, toughness, and tensile performance, accelerating premature cracking and shifting fracture behavior from ductile to brittle modes [69,70,71].

## 4. Conclusions

In this study, the fabrication of Cu–SiC metal matrix composites was demonstrated through a hybrid approach combining powder metallurgy with thermo-compression processing. The deformation–annealing sequence proved effective in reducing porosity, refining grains, and strengthening interfacial bonding, thereby overcoming the limitations of conventionally sintered composites.

The primary outcomes can be summarized as follows:

1. Influence of SiC content: Low-to-moderate reinforcement levels (1–3 wt.% SiC) supported high deformation (approximately 60–68%) and effective densification. Higher SiC contents caused brittleness, particle agglomeration, and reduced deformability.
2. Corrected interpretation of optimum performance: The Cu–3wt.% SiC composite exhibited the highest mechanical performance not because it possessed the lowest porosity, but due to its uniform reinforcement dispersion, improved particle–matrix bonding, and stable strain hardening behavior.
3. Effectiveness of thermo-compression: The TCP substantially reduced pore size, increased density, and improved interfacial bonding through pore collapse and grain refinement. These effects were consistent across all compositions but were most beneficial in Cu–rich matrices.
4. The thermo-compression process increased the UTS from 71 to 146 MPa in pure Cu and identified ~3 wt.% SiC as the optimum reinforcement level, achieving ~209 MPa UTS, ~65 HRB hardness, and ~35 MJ/m^3^ toughness with the best balance of strength and ductility.
5. Limitation at high reinforcement levels: The Higher the SiC contents (≥5 wt.%), the restricted plastic flow, increased brittleness, and decreased toughness despite improved hardness, indicating that excessive ceramic content is detrimental to overall structural performance.
6. Oxidation influence: Minor Cu_2_O/CuO formation affected local fracture behavior by promoting particle pull-out and localized embrittlement, particularly in undeformed samples.

In summary, the integrated powder metallurgy and the thermo-compression route significantly enhanced densification and mechanical performance. Reinforcement levels of 1–3 wt.% SiC was found to provide the most balanced combination of strength, ductility, and microstructural stability for Cu–SiC metal matrix composites.

## Figures and Tables

**Table 1:** Composition of Cu–SiC Composite Samples with Varying Reinforcement Content.

Sr. No.	Sample	Weight Percentage of Matrix and Reinforcing Agent	
		Copper (wt.%)	SiC (wt.%)
1	A	100	-
2	B	99	1
3	C	98	2
4	D	97	3
5	E	96	4
6	F	95	5
7	G	90	10
8	H	85	15
9	I	80	20
10	J	75	25

**Table 2:** Thermo-compression processing sequence applied to Cu–SiC composites.

Step	Process	Condition/Parameter	Purpose
1	Initial Compaction	150 MPa, room temperature	Form a green pellet
2	Sintering	950 °C, 60 min; Argon atmosphere	Densification, bonding
3	Deform 1	Open-die cold forging	~20% height reduction
4	Annealing 1	450 °C, 60 min, Ar	Stress relief & recovery
5	Deform 2	Open-die cold forging	~25% height reduction
6	Annealing 2	450 °C, 60 min, Ar	Stress relief & recovery
7	Deform 3	Open-die cold forging	~20% reduction
8	Annealing 3	450 °C, 60 min, Ar	Remove residual work hardening
9	Deform 4	Open-die cold forging	~30% reduction
10	Final Annealing	Optional: 450 °C, 60 min, Ar	Final stress relief

**Table 3:** Deformation parameters and specimen height after each deformation stage (Deform 1–Deform 4) during open-die cold forging of Cu–SiC composites.

Composite	Initial Height (mm)	Deform 1 (mm)	Deform 2 (mm)	Deform 3 (mm)	Deform 4 (mm)	Percentage Deformation
Cu	5.84	4.67	3.50	2.80	1.96	66.44
Cu–1wt.% SiC	5.78	4.62	3.47	2.77	1.85	67.99
Cu–2wt.% SiC	5.85	4.68	3.51	2.81	2.08	64.44
Cu–3wt.% SiC	5.95	4.76	3.57	2.86	2.19	63.19
Cu–4wt.% SiC	6.03	4.82	3.62	2.89	1.96	67.50
Cu–5wt.% SiC	6.25	5.00	3.75	3.00	2.54	59.36
Cu–10wt.% SiC	6.96				3.72	46.55
Cu–15wt.% SiC	8.08				5.55	31.31
Cu–20wt.% SiC	9.24				8.5	8.01
Cu–25wt.% SiC	10.12				9.99	1.28

**Table 4:** Density and porosity of Cu–SiC composites with varying SiC content.

Composite	Green Density (g/cm^3^)	Sintered Density (g/cm^3^)	Relative Density (%)	Initial Porosity (%)
Cu	7.1	8.7	97.3	11.7
Cu–1wt.% SiC	7.3	8.5	96.4	13.5
Cu–2wt.% SiC	7.0	7.4	94.7	14.1
Cu–3wt.% SiC	8.0	7.3	90.3	15.2
Cu–4wt.% SiC	6.7	7.2	88.9	17.3
Cu–5wt.% SiC	6.9	7.1	88.4	20.6

**Table 5:** Mechanical properties of Cu–SiC composites before and after the deformation.

Composite		Density	Yield Stress	Ultimate Tensile Strength	Toughness	Fracture Strength	Elongation at Break
		g/cm^3^	MPa	MPa	KJ/m^3^	MPa	(%)
Cu	Undeformed	8.15	45.5	71	2.5	68	7.1
	Deformed	8.71	40.4	145.7	10.3	121.3	12
Cu–1wt.% SiC	Undeformed	7.6	107.4	166	24.2	165	27.2
	Deformed	8.47	95.9	193	35.9	152	23.1
Cu–3wt.% SiC	Undeformed	7.26	75.1	283	25.6	85	16
	Deformed	8.09	51.9	208.8	35.1	71	24.6
Cu–5wt.% SiC	Undeformed	6.98	34	104	7.1	93	11.8
	Deformed	7.58	35.4	147	8.8	205	9.4

## Data Availability

The original contributions presented in the study are included in the article/Supplementary Materials, further inquiries can be directed to the corresponding authors.

## Supplementary Materials

The following supporting information can be downloaded at: https://www.mdpi.com/article/10.3390/ma19020243/s1.
