# Supplementary material for: Tailoring Microstructure and Performance of Cu/SiC Composites via Integrated Powder Metallurgy and Thermo-Compression Processing
Source: Materials (Basel). 2026 Jan 7;19(2):243. doi: 10.3390/ma19020243 (PMC12843209; doi:10.3390/ma19020243)
Supplement: Supplementary file 1 [file materials-19-00243-s001.zip › materials-4011013-supplementary.pdf]

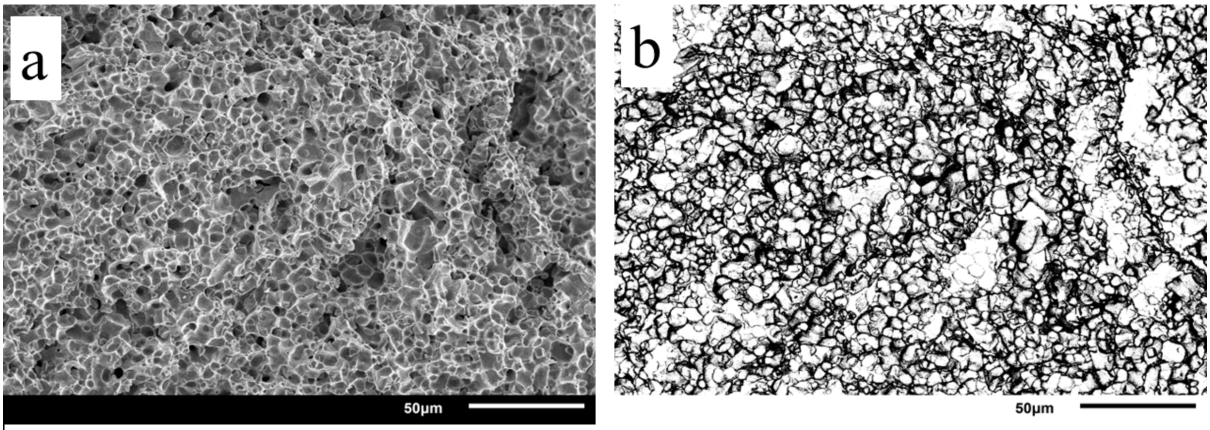

Figure S1 (a) SEM image of pure Cu (undeformed). (b) ImageJ-processed binary output used for pore quantification (scale bar: 50 μm).

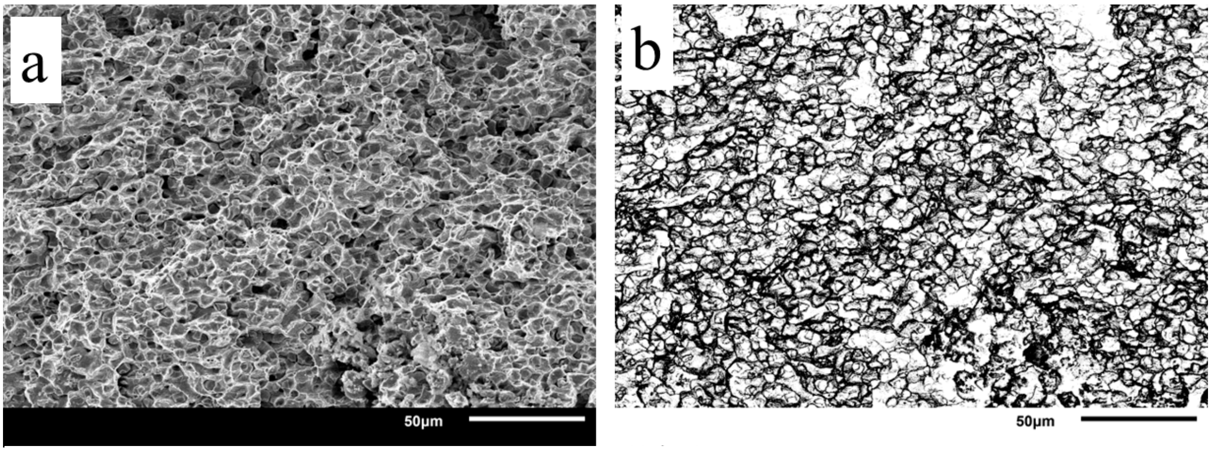

Figure S2 (a) SEM image of pure Cu (deformed). (b) ImageJ-processed binary output used for pore quantification (scale bar: 50 μm).

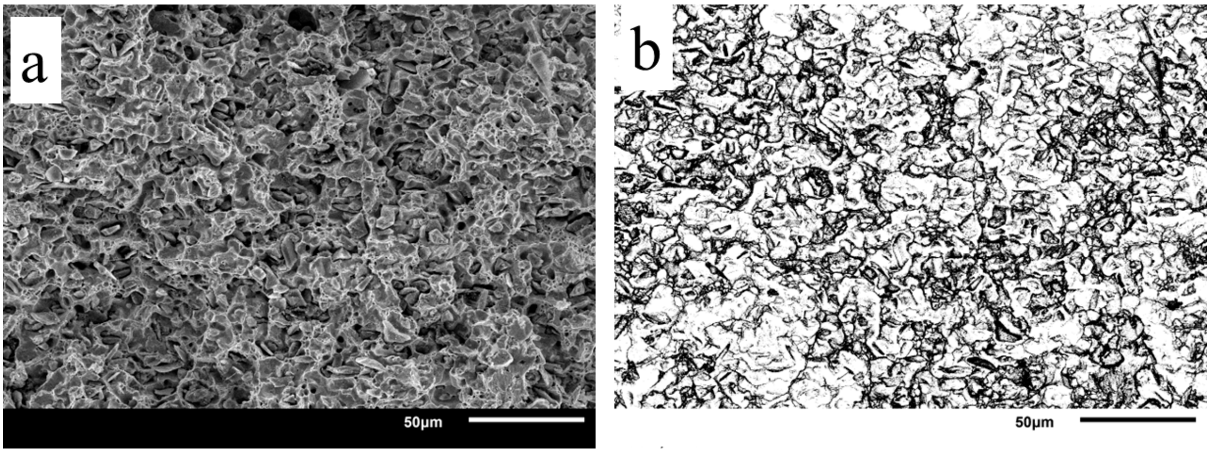

Figure S3 SEM image of the undeformed Cu–SiC composite. (b) ImageJ-processed binary output used for pore quantification (scale bar: 50 μm).

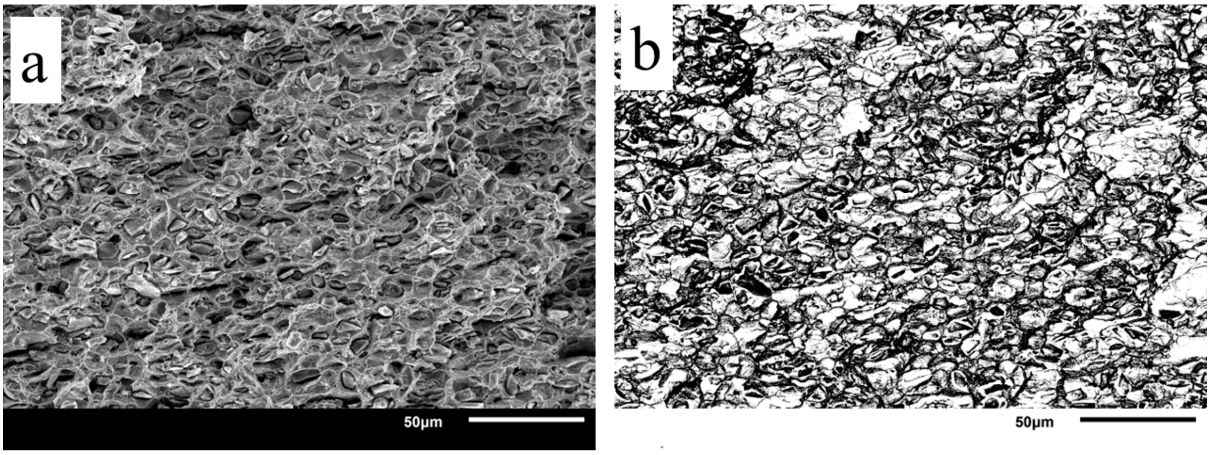

Figure S4 (a) SEM image of the deformed Cu–SiC composite. (b) ImageJ-processed binary output used for pore quantification (scale bar: 50 μm).
